# Supplementary material for: Development and validation of risk prediction model for diabetic neuropathy among diabetes mellitus patients at selected referral hospitals, in Amhara regional state Northwest Ethiopia, 2005–2021
Source: PLoS One. 2023 Aug 29;18(8):e0276472. doi: 10.1371/journal.pone.0276472 (PMC10465000; doi:10.1371/journal.pone.0276472)
Supplement: S1 File — (DOCX) [file pone.0276472.s001.docx]

Supplementary files

S1: Missing variables and its percentage for development and validation of risk prediction model for diabetic neuropathy among diabetes mellitus patients at selected referral hospitals, in Amhara regional state Northwest Ethiopia, 2005-2021.,

| Missing variable | Missing value(no.) | Missing  Value (%) | Missing variable | Missing value(no.) | Missing value (%) |
| --- | --- | --- | --- | --- | --- |
| Marital status | 710 | 87.87 | Body mass index | 400 | 49.5% |
| Religion | 706 | 87.38 | Hypertension | 78 | 9.65 |
| Education | 799 | 98.89 | MABP | 13 | 1.61 |
| Occupation | 782 | 96.78 | DM complication | 602 | 74.5 |
| Family history | 740 | 91.58 |  |  |  |
| Surgical history | 780 | 96.5 | Other comorbidities | 95 | 11.6 |
| Psychiatric history | 794 | 98.2 | Adherence | 163 | 20 |
| Drug allergy | 796 | 98.5 | Glycemic control | 17 | 2.1 |
| HbA1c | 615 | 76.1 | Hemoglobin | 384 | 47.5 |
| BUN | 703 | 87. | TG | 327 | 40 |
| Uric acid | 792 | 98 | TC | 313 | 38 |
| Albumin | 799 | 98 | Creatinine | 199 | 24.6 |
| RBS | 683 | 84 | WBC | 398 | 48 |
| **Complete cases** |  |  | RBC | 391 | 49 |
| Baseline age | 0 | 0 |  |  |  |
| Sex | 0 | 0 | Platelets | 399 | 48 |
| Residence | 0 | 0 | Physical activity | 386 | 47 |
| Type of DM | 0 | 0 | Smoking | 615 | 76.1 |
| Type of treatment | 0 | 0 | Alcohol drinking | 361 | 44 |
| Duration | 0 | 0 | Unhealthy diet | 381 | 47 |
