# Supplementary material for: Development and validation of risk prediction model for diabetic neuropathy among diabetes mellitus patients at selected referral hospitals, in Amhara regional state Northwest Ethiopia, 2005–2021
Source: PLoS One. 2023 Aug 29;18(8):e0276472. doi: 10.1371/journal.pone.0276472 (PMC10465000; doi:10.1371/journal.pone.0276472)
Supplement: S2 File — (DOCX) [file pone.0276472.s002.docx]

S2: - Predictive performance of for individual and combined predictors by AUC value for development and validation of risk prediction model for diabetic neuropathy among diabetes mellitus patients at selected referral hospitals, in Amhara regional state Northwest Ethiopia, 2005-2021.

| Predictors | AUC (95% CI) |
| --- | --- |
| Glycemic control | 0.544(0.513.0.575) |
| Comorbidities | 0.648(0.608, 0.686) |
| Physical activity | 0.527(0.485,0.568) |
| Harmful use of alcohol | 0.511(0.469,0.552) |
| Hypertension | 0.596(0.554,0.637) |
| Type of treatment | 0.583(0.540,0.626) |
| WBC count | 0.542(0.499,0.583) |
| RBC count | 0.536(0.492,0.580) |
| GC+ Comorbidities | 0.662(0.621,0.722) |
| GC+ Physical activity+ comorbidities | 0.69.3(0.648,0.734) |
| GC+ Physical activity+ WBC+ comorbidities | 0.698(0.654,0.742) |
| GC+ Physical activity+ RBC+ WBC+ comorbidities | 0.708(0.661,0.754) |
| GC+ physical activity+ RBC+WBC+ Comorbidities+ HTN | 0.716(0.673,0.760) |
| GC+HTN+ comorbidities+ physical activity+ alcohol+ RBC+ WBC | 0.725(0.682,0.768) |
| GC+ HTN+ Comorbidities+ Physical activity+ alcohol+ RBC+ WBC+ Treatment type | 0.732(0.690,0.773) |
