# Supplementary material for: Development and validation of risk prediction model for diabetic neuropathy among diabetes mellitus patients at selected referral hospitals, in Amhara regional state Northwest Ethiopia, 2005–2021
Source: PLoS One. 2023 Aug 29;18(8):e0276472. doi: 10.1371/journal.pone.0276472 (PMC10465000; doi:10.1371/journal.pone.0276472)
Supplement: Attachment — Submitted filename: Point by point response for editors.docx [file pone.0276472.s003.docx]

**Point by point response for editors/reviewers comments**

**PLOSE ONE Journal**

**Manuscript title:** **Prediction of the risk for diabetic neuropathy among diabetes mellitus patients at selected referral hospitals, in Amhara regional state Northwest Ethiopia, January, 2005- December 2021; risk prediction model development and validation**

**Manuscript ID:**  PONE-D-22-27723

Dear editor/reviewer, we would like to thank you for the constructive, building, and improvable comments on this manuscript that would improve the content of the manuscript. We considered each comment and clarification question of editors and reviewers on the manuscript thoroughly. Our point-by-point responses for each comment and question are described in detail on the following pages. Further, the details of changes were shown by track changes in the supplementary document attached.

**Response to Editor**

1. Please ensure that your manuscript meets PLOS ONE's style requirements, including those for file naming. The PLOS ONE style templates can be found at <https://journals.plos.org/plosone/s/file?id=wjVg/PLOSOne_formatting_sample_main_body.pdf> and
   <https://journals.plos.org/plosone/s/file?id=ba62/PLOSOne_formatting_sample_title_authors_affiliations.pdf>

**Authors’ response:** Thank you Editor. We have modified the manuscript as per the PLOS ONE journal style requirements. **(See the revised manuscript)**

2. Please provide additional details regarding participant consent. In the ethics statement in the Methods and online submission information, please ensure that you have specified (1) whether consent was informed and (2) what type you obtained (for instance, written or verbal, and if verbal, how it was documented and witnessed). If your study included minors, state whether you obtained consent from parents or guardians. If the need for consent was waived by the ethics committee, please include this information. If you are reporting a retrospective study of medical records or archived samples, please ensure that you have discussed whether all data were fully anonymized before you accessed them and/or whether the IRB or ethics committee waived the requirement for informed consent. If patients provided informed written consent to have data from their medical records used in research, please include this information.

**Authors’ response:** In ethical statement, Since we use secondary data source that is medical records from two selected referral hospitals without contacting to patients but for medical records “Ethical clearance/ statement” were got from institute of public health, university of Gondar Ethical review board and the letter attached to as supplementary files. Therefore, It is waived and had no harm on study participates rather it is useful for decision making for intervention to prevent diabetic neuropathy and further complication.

For medical records the IRB waived the requirement for written or verbal consent since no contact to patients rather the Ethics committee given ethical clearance to do the research by confirming that no harm to patients and the letter attached in supplementary files.

**Comments to the Author**

1. Is the manuscript technically sound, and do the data support the conclusions? The manuscript must describe a technically sound piece of scientific research with data that supports the conclusions. Experiments must have been conducted rigorously, with appropriate controls, replication, and sample sizes. The conclusions must be drawn appropriately based on the data presented.

Reviewer #1: Partly

Author response; I was try to correct in the revised manuscript with appropriate Plos one journal requirements.

2. Has the statistical analysis been performed appropriately and rigorously?

Reviewer #1: Yes

3. Have the authors made all data underlying the findings in their manuscript fully available? The PLOS Data policy requires authors to make all data underlying the findings described in their manuscript fully available without restriction, with rare exception (please refer to the Data Availability Statement in the manuscript PDF file). The data should be provided as part of the manuscript or its supporting information, or deposited to a public repository. For example, in addition to summary statistics, the data points behind means, medians and variance measures should be available. If there are restrictions on publicly sharing data—e.g. participant privacy or use of data from a third party—those must be specified.

Reviewer #1: Yes

4. Is the manuscript presented in an intelligible fashion and written in standard English? PLOS ONE does not copyedit accepted manuscripts, so the language in submitted articles must be clear, correct, and unambiguous. Any typographical or grammatical errors should be corrected at revision, so please note any specific errors here.

Reviewer #1: Yes

5. Review Comments to the Author Please use the space provided to explain your answers to the questions above. You may also include additional comments for the author, including concerns about dual publication, research ethics, or publication ethics. (Please upload your review as an attachment if it exceeds 20,000 characters)

Reviewer #1: Negalgn Byadgie Gelaw et al. investigated the prediction of the risk for diabetic nephropathy in Ethiopia. In multivariable logistic regression, glycemic control, physical activity, hypertension, alcohol drinking, type of treatment, white blood cells and red blood cells count were statistically significant. Nomogram and mobile based tool were developed and AUC, discrimination performance by bootstrapping method, and classification and regression performance by machine learning were 73.2%, 71.7%, and 70.2%, respectively.

**Author response**; I was correct all ethical issues, publication issues

**Major comments**

1. . In lines 241-246, the complete diagnostic criteria should be presented. The thresholds of each diagnostic component should be shown. How many items should be positive for the diagnosis of diabetic neuropathy? What was the diagnostic criteria for the description of diabetic neuropathy in medical record?

**Authors response**; we modified as your comment in revised manuscripit in line 241-246 the complete diagnostic criteria for diabetic neuropathy was different corrected in operational definition.

We use clinical criteria and confirmed by different tests, since different type of neuropathies.

The typical clinical sign and symptoms is numbness on hands and feet’s, pain, tingling, paraesthesia , gait ataxia and confirmed by pinprick and temperature examination, touch sensation by 10g monofilament, vibration sense by biothesiometer and ankle reflex

In medical records, the medical doctors written as diabetic neuropathy after they took detailed clinical history, physical examination with advanced imaging technology.

2. In lines 161-179, the design of clinical study design is not well described. Since the authors predict the risk for diabetic neuropathy, they should perform the retrospective cohort study. The authors should describe the exposures and observation periods.

**Authors response;** We were correct as in line 161-169 the design of the study is retrospective follow up study since our interest is follow the exposed groups (Diabetes mellitus patients) for 16 years to identify who is at risk of developing diabetic neuropathy and develop clinical model based on the data got from 808 DM patients for early diagnosis and applying early intervention to prevent further complication and death

3. In page 10 and Table 1, the authors should describe the definition and thresholds of “rural vs urban”, “alcohol drinking”, “physical activity” and “unhealthy diet”.

**Authors response;** The operation definition and threshold of alcohol drinking, physical activity and unhealthy diet were correct and written in operational definition section

4.In page 11 and Table 2, did the patients receive the GLP-1 receptor agonists? GLP-1 receptor agonists may be beneficial for the diabetic neuropathy

**Authors response;** After diagnosing DM, the patients took GLP-1 agonists medication to prevent diabetic neuropathy and other DM complication such as; retinopathy, nephropathy

5. In lines 408-411, the formula can be applied for the subjects with normal glucose tolerance (NGT), impaired fasting glucose (IFG), and impaired glucose tolerance (IGT)? The authors should use the continuous values rather than descreted values for the clinical parameters.

**Authors response;** The clinical model formula used for all confirmed/diagnosed Diabetes mellitus patients with or without normal glucose tolerance to identify low or high risk of DM patients to develop diabetic neuropathy. If low risk, then counsel to try to keep their health condition or if high risk to try to improve their health condition by taking drugs accordingly , avoid eating fat contain foods, do physical exercise. Therefore, the formula is very important for all DM patients to predict diabetic neuropathy.

Using clinical parameters as continuous is recommended to prevent loss of information but fore sake of developing mobile based tool and easily applicability, we categorized based on the standard criteria.

**Minor comments**

1. In lines 79, 97, and 138, “burdon” should be “burden”.

**Authors response;** We were correct it in line 79,97,138 as burden

6.PLOS authors have the option to publish the peer review history of their article (what does this mean?). If published, this will include your full peer review and any attached files. If you choose “no”, your identity will remain anonymous but your review may still be made public. Do you want your identity to be public for this peer review? For information about this choice, including consent withdrawal, please see our Privacy Policy.

Reviewer #1: No

Thank you so much for your kind support!!
