# Supplementary material for: Development and validation of risk prediction model for diabetic neuropathy among diabetes mellitus patients at selected referral hospitals, in Amhara regional state Northwest Ethiopia, 2005–2021
Source: PLoS One. 2023 Aug 29;18(8):e0276472. doi: 10.1371/journal.pone.0276472 (PMC10465000; doi:10.1371/journal.pone.0276472)
Supplement: Attachment — Submitted filename: Response to Reviewers.docx [file pone.0276472.s004.docx]

**Response for editors/reviewers comments**

**PLOSE ONE Journal**

**Manuscript title:**  **Development and validation of risk prediction model for diabetic neuropathy among diabetes mellitus patients at selected referral hospitals, in Amhara regional state Northwest Ethiopia, 2005-2021.**

**Manuscript ID:**   PONE-D-22-27723R1

Dear editor/reviewer, we would like to thank you for the constructive, building, and improvable comments on this manuscript that would improve the content of the manuscript. We considered each comment and clarification question of editors and reviewers on the manuscript thoroughly. Our point-by-point responses for each comment and question are described in detail on the following pages. Further, the details of changes were shown by the manuscript with track changes

**Response to academic editor and reviewer(s)**

**Journal Requirements:**

Please review your reference list to ensure that it is complete and correct. If you have cited papers that have been retracted, please include the rationale for doing so in the manuscript text, or remove these references and replace them with relevant current references. Any changes to the reference list should be mentioned in the rebuttal letter that accompanies your revised manuscript. If you need to cite a retracted article, indicate the article’s retracted status in the References list and also include a citation and full reference for the retraction notice.

**Authors’ response**: Thank you Editor. The retracted article reference that we use in our manuscript is updated, enough evidence and appropriate reports which is useful for providing information for current situation and predicting for future. For example National Strategic Action Plan report prepared by federal ministry of health in Ethiopia and other books like handbook of clinical epidemiology used for prediction modeling

**Reviewers' comments:**

**Reviewer's Responses to Questions**

**Comments to the Author**

1. If the authors have adequately addressed your comments raised in a previous round of review and you feel that this manuscript is now acceptable for publication, you may indicate that here to bypass the “Comments to the Author” section, enter your conflict of interest statement in the “Confidential to Editor” section, and submit your "Accept" recommendation.

**Reviewer #1: All comments have been addressed**

2. Is the manuscript technically sound, and do the data support the conclusions? The manuscript must describe a technically sound piece of scientific research with data that supports the conclusions. Experiments must have been conducted rigorously, with appropriate controls, replication, and sample sizes. The conclusions must be drawn appropriately based on the data presented.

**Reviewer #1: Yes**

3. Has the statistical analysis been performed appropriately and rigorously?

**Reviewer #1: Yes**

4. Have the authors made all data underlying the findings in their manuscript fully available? The PLOS Data policy requires authors to make all data underlying the findings described in their manuscript fully available without restriction, with rare exception (please refer to the Data Availability Statement in the manuscript PDF file). The data should be provided as part of the manuscript or its supporting information, or deposited to a public repository. For example, in addition to summary statistics, the data points behind means, medians and variance measures should be available. If there are restrictions on publicly sharing data—e.g. participant privacy or use of data from a third party—those must be specified.

**Reviewer #1: Yes**

5. Is the manuscript presented in an intelligible fashion and written in standard English? PLOS ONE does not copyedit accepted manuscripts, so the language in submitted articles must be clear, correct, and unambiguous. Any typographical or grammatical errors should be corrected at revision, so please note any specific errors here.

**Reviewer #1: No**

**Authors’ response:** We have modified the manuscript as per the PLOS ONE journal style requirements on intelligible fashion and written in Standard English. **(See the revised manuscript with track changes)**

6. Review Comments to the Author Please use the space provided to explain your answers to the questions above. You may also include additional comments for the author, including concerns about dual publication, research ethics, or publication ethics. (Please upload your review as an attachment if it exceeds 20,000 characters)

Reviewer #1: Authors response: After diagnosing DM, the patients took GLP-1 agonists medication to prevent diabetic neuropathy and other DM complication such as; retinopathy, nephropathy. Please describe the percentage of the patients treated with the GLP-1 receptor agonists in Table 2.

**Authors’ response:** Although the GLP-1 receptor agonists currently approved in the United States for the treatment of type 2 diabetes include exenatide , liraglutide and lixisenatide , albiglutide, and dulaglutide. But the above drugs is not available in our set up or study area/selected referral hospitals/. Instead of that especially for type-2 diabetes mellitus patients 460(56.9%) took metformin oral drug.

7. PLOS authors have the option to publish the peer review history of their article (what does this mean?). If published, this will include your full peer review and any attached files. If you choose “no”, your identity will remain anonymous but your review may still be made public. Do you want your identity to be public for this peer review? For information about this choice, including consent withdrawal, please see our Privacy Policy.

**Reviewer #1: No**

[NOTE: If reviewer comments were submitted as an attachment file, they will be attached to this email and accessible via the submission site. Please log into your account, locate the manuscript record, and check for the action link "View Attachments". If this link does not appear, there are no attachment files.]

While revising your submission, please upload your figure files to the Preflight Analysis and Conversion Engine (PACE) digital diagnostic tool, https://pacev2.apexcovantage.com/. PACE helps ensure that figures meet PLOS requirements. To use PACE, you must first register as a user. Registration is free. Then, login and navigate to the UPLOAD tab, where you will find detailed instructions on how to use the tool. If you encounter any issues or have any questions when using PACE, please email PLOS at figures@plos.org. Please note that Supporting Information files do not need this step.

**Author response;** we upload our figure files to the Preflight Analysis and Conversion Engine (PACE) digital diagnostic tool and eligible for PLOS ONE.
